# Regulation of the retinoblastoma proteins by the human herpesviruses

**DOI:** 10.1186/1747-1028-4-1

**Published:** 2009-01-15

**Authors:** Adam J Hume, Robert F Kalejta

**Affiliations:** 1 Institute for Molecular Virology and McArdle Laboratory for Cancer Research, University of Wisconsin-Madison, Madison, WI 53706-1596, USA

## Abstract

Viruses are obligate intracellular parasites that alter the environment of infected cells in order to replicate more efficiently. One way viruses achieve this is by modulating cell cycle progression. The main regulators of progression out of G0, through G1, and into S phase are the members of the retinoblastoma (Rb) family of tumor suppressors. Rb proteins repress the transcription of genes controlled by the E2F transcription factors. Because the expression of E2F-responsive genes is required for cell cycle progression into the S phase, Rb arrests the cell cycle in G0/G1. A number of viral proteins directly target Rb family members for inactivation, presumably to create an environment more hospitable for viral replication. Such viral proteins include the extensively studied oncoproteins E7 (from human papillomavirus), E1A (from adenovirus), and the large T (tumor) antigen (from simian virus 40). Elucidating how these three viral proteins target and inactivate Rb has proven to be an invaluable approach to augment our understanding of both normal cell cycle progression and carcinogenesis. In addition to these proteins, a number of other virally-encoded inactivators of the Rb family have subsequently been identified including a surprising number encoded by human herpesviruses. Here we review how the human herpesviruses modulate Rb function during infection, introduce the individual viral proteins that directly or indirectly target Rb, and speculate about what roles Rb modulation by these proteins may play in viral replication, pathogenesis, and oncogenesis.

## Background

The members of the retinoblastoma family of tumor suppressors, Rb, p107 and p130, are transcriptional co-repressors that regulate both differentiation and cell cycle progression. Detailed reviews on genetic and molecular analysis of the Rb pathway in normal and cancerous cells are numerous [1-6] so here we will only briefly introduce this pathway prior to describing how it is manipulated by the human herpesviruses.

In the G0 and G1 phases of the cell cycle the active, hypophosphorylated form of Rb binds to transcription factors of the E2F family [7-9]. Through heterodimerization with the DP proteins, E2Fs bind to promoters and control the transcription of genes that are involved in many important cellular functions including cell cycle progression [2,10], DNA replication [11,12], the DNA damage response [13], apoptosis [14-18], differentiation and development [19-21], senescence [22], and angiogenesis [23]. Rb, which itself is an E2F-responsive gene [24], binds to E2Fs at these promoters to actively repress transcription by blocking the E2F activation domain, and by recruiting histone-modifying enzymes such as histone deacetylases (HDACs) [25-28] and chromatin remodeling proteins such as the members of the hSWI/SNF complex [29-31]. There are multiple E2F proteins, some considered mainly as transcriptional repressors, and others with more prominent roles as transcriptional activators.

Many cellular and viral proteins interact with the pocket domain of Rb [32] that consists of A and B subdomains and affords a large surface area to support strong and specific interactions. One common amino acid sequence found in proteins that interact with the Rb pocket is the LxCxE motif [25,26,33-35]. More than twenty cellular proteins such as HDACs, and a number of viral proteins interact with Rb in an LxCxE-dependent manner [35-37]. The Rb-binding LxCxE motif interacts with a site within the Rb pocket termed the cleft region. The E2F proteins do not contain LxCxE motifs and bind to a distinct site within the Rb pocket domain, allowing Rb to interact with both an E2F and HDAC simultaneously to repress transcription of E2F-responsive promoters.

HDAC-Rb-E2F complexes are disrupted during the natural progression of cells out of G0, through G1, and into the S phase. In a sequential and coordinated fashion, different cyclin proteins are expressed and they bind to and activate a family of cyclin-dependent kinases (Cdks). Certain cyclin/Cdk complexes control cell cycle progression, whereas others modulate the function of the RNA Polymerase II transcriptional complex [38,39]. Small molecule inhibitors of Cdk activity often inhibit multiple members of both the "cell cycle" Cdks and the "transcription" Cdks [40,41], so prescribing the effects of these inhibitors solely to modulation of cell cycle processes must be done judiciously. The Rb protein is one of the main "cell cycle" Cdk substrates, containing 16 putative Cdk phosphorylation sites. Hyperphosphorylation of Rb by a series of cyclin/Cdk pairs causes the disruption of HDAC-Rb-E2F complexes [2,5,6,10,30,42-45], allowing for the activation of E2F-responsive genes and the subsequent progression of cells through G1 and into the S phase. Phosphorylation at any one site is insufficient to disassemble complexes between Rb and its binding proteins, whereas the accumulation of multiple phosphorylations appears to be necessary for complex disruption [43,44].

Cyclins D, E and A, in that order, are the targeting modules that direct the Cdks to phosphorylate different subsets of Cdk consensus sites on Rb. The D-type cyclin proteins have both an LxCxE motif and a region termed the hydrophobic patch that contribute to both substrate and substrate site specificity [46-48]. Cyclins E and A contain a hydrophobic patch but lack LxCxE motifs. As mentioned above, the LxCxE motif binds within the cleft domain of Rb [34,37]. The hydrophobic patch binds to RxL motifs located within the C-terminus of Rb [49].

Because the phosphorylation of Rb by cyclin/Cdks represents a critical juncture in the control of cell cycle progression, this is a tightly regulated process. Multiple levels of regulation exist that include temporal cyclin expression, activating and inhibitory phosphorylation of Cdks, and the direct binding and inhibition of cyclin/Cdk complexes by two classes of small proteins termed the cyclin-dependent kinase inhibitors (Ckis) [50,51]. Additionally, Rb must return to a hypophosphorylated form during mitosis so that the cell cycle can be reset. This is achieved by the action of protein phosphatase I (PPI) [52-54]. Although there is little evidence that Rb is regulated by degradation during normal cell cycle progression, the over expression of the cellular protein gankyrin can result in Rb degradation [55] through a process that requires an intact LxCxE motif of gankyrin [56].

The pathway controlled by Rb is thought to be inactivated in most if not all human cancers [2,57]. Common mechanisms of pathway disruption include the over-expression and stabilization of cyclin D [1,58,59], inactivation of the Cki p16 [60,61], or the expression of a viral oncoprotein [62]. Certain cancers also have activating Cdk mutations [63,64], Rb loss or mutation [65], or gankyrin over-expression [55]. The other members of the Rb family, p107 and p130, also regulate E2F-mediated gene expression and are targets of the Cdks. But whereas Rb is present throughout the cell cycle, p107 and p130 each have a more limited window of expression. The p130 protein is found predominantly in G0 cells [66,67], is rapidly phosphorylated upon entry into G1 [66,68], and swiftly degraded following phosphorylation [69]. The p107 protein is expressed as cells begin to enter the S phase [67,70]. While Rb is often mutated in human cancers and thus is considered a true tumor suppressor protein, p130 is infrequently mutated [71-76], and no p107 mutations in human cancers have been identified.

### DNA tumor viruses inactivate Rb

Cells must synthesize large amounts of DNA to replicate their genomes during the cell division cycle. Both enzymes that synthesize or metabolize deoxynucleotides, as well as enzymes that directly or indirectly facilitate deoxynucleotide polymerization are required for DNA synthesis. The coordinated production of these required enzymes is achieved by placing them under the control of the E2F transcription factors that in turn are regulated by Rb. Because viruses with DNA genomes must also synthesize significant quantities of this nucleic acid during their productive, lytic replication phases, many have evolved ways to modulate the Rb-E2F pathway. Viral inactivation of Rb has been most extensively studied through examining the relevant transforming oncoproteins of the DNA tumor viruses, namely the Adenovirus E1A protein, the Papillomavirus E7 protein, and the Simian Virus 40 (SV40) large tumor (T) antigen. Many excellent reviews of these proteins have been published [77-79]. While the focus of this review is herpesvirus proteins that modulate the Rb pathway, a brief description of the DNA tumor virus proteins that also accomplish this task is presented to provide a platform for comparison and contrast.

Adenovirus E1A contains an LxCxE motif (the conserved region 2 (CR2) domain) that interacts in the Rb cleft [80-82], and a second motif (CR1) that competes with E2F for binding to Rb [80-82]. In this manner, E1A disrupts complexes between the E2Fs and all three Rb family members. E1A expression stimulates cell cycle progression and cooperates with other viral or cellular oncogenes to transform cells. For many years no association between adenoviral infection and human cancers could be identified, but a recent study has observed a link between fetal adenoviral infections and childhood acute lymphoblastic leukemia [83].

The human papillomavirus encoded E7 also contains an LxCxE motif, binds to all three Rb proteins, and induces their degradation through the ubiquitin-proteasome pathway [84-88]. In addition to the LxCxE domain, N-terminal sequences of unknown function are required for E7-induced Rb degradation [89]. The exact mechanism of degradation has not yet been determined. It was initially speculated that E7-mediated degradation occurred through a direct interaction with the proteasome because E7 was found to bind to the S4 subunit of the 26S proteasome [90]. However, E7 mutants that fail to bind S4 still induce Rb degradation [87]. Recently, E7 has been found to associate with an active cullin 2-based cellular ubiquitin ligase complex [91], thus it is possible that E7 redirects this cellular complex to ubiquitinate Rb. While infection with some papillomavirus genotypes (termed high risk) can lead to cervical cancer, infection with other genotypes (low risk) does not [87]. The E7 proteins from high risk papillomavirus subtypes efficiently bind and degrade Rb, and cooperate with other viral or cellular oncoproteins to transform cells [87]. E7 proteins from low risk HPV fail to transform cells but still bind and degrade Rb, although with lower efficiency than the high risk HPV E7 proteins [92].

SV40 T Antigen contains an LxCxE motif, binds to all three Rb proteins [93-95], and disrupts Rb-E2F complexes [96-98]. An additional T antigen domain with sequence homology to the cellular chaperone DNA J is also required for T antigen inactivation of all three Rb proteins [99,100], and for dephosphorylation of p107 and p130 [98,100,101]. The role of T antigen-mediated dephosphorylation of p107 and p130 is not understood. SV40 has never been definitively associated with human tumors, although studies continue to examine a potential role for SV40 infection via contaminated polio vaccines in mesotheliomas [99,102,103].

**Figure 1:**
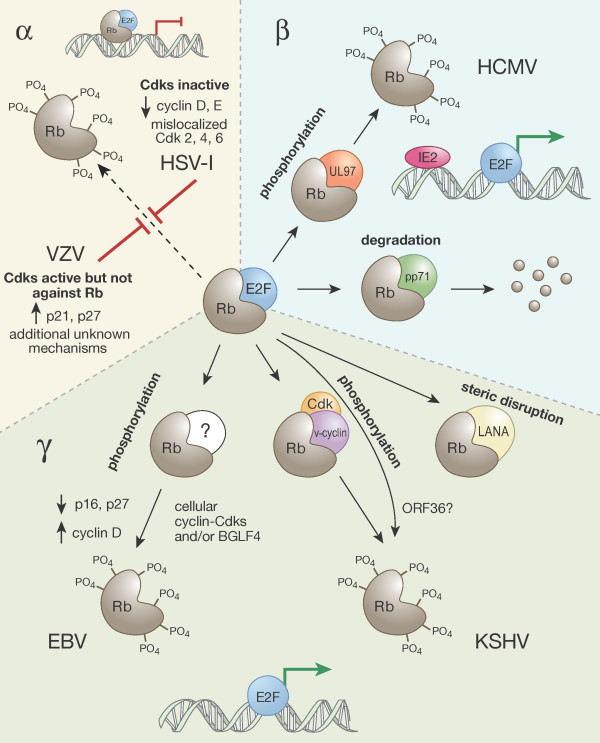
**Mechanisms used by different classes of human herpesviruses to modulate the Rb-E2F pathway**. See text for details. **α**. HSV-1 prevents Rb phosphorylation and keeps cellular Cdks inactive, likely through the downregulation of cyclin proteins and the mislocalization of Cdks. VZV infected cells display Cdk activity even though p21 and p27 are induced. However, Rb remains unphosphorylated through an uncharacterized mechanism.**β**. HMCV infected cells show a lack of hypophosphorylated Rb and high levels of hyperphosphorylated Rb, due to both pp71-mediated degradation and UL97-mediated phosphorylation. IE2 can also directly activate E2F-responsive genes.**γ**. EBV has multiple proteins (Z, R, LMP-1, EBNA-2,-3C,-5) that could lead to the phosphorylation of Rb by cellular Cdks, and/or may directly phosphorylate Rb thropugh the function of the viral kinase, BGLF-4. KSHV can activate the Rb pathway by LANA-mediated disruption of Rb-E2F complexes, or by direct phosphorylation of Rb through the action of the v-cyclin and/or the ORF36 proteins.

These three viral proteins represent two distinct mechanisms of Rb inactivation: steric disruption of Rb-E2F complexes and Rb degradation. Herpesviruses encode proteins that use these, as well as other novel (direct and indirect) mechanisms to inhibit Rb family member function (Figure 1). The study of E1A, E7, and T Antigen has proven to be invaluable for the understanding of both the replication and pathogenesis of the viruses that encode them, as well as to the normal regulation of the Rb-E2F pathway. The study of herpesvirus-encoded proteins that modulate Rb through novel mechanisms should therefore also lead to a better understanding of both herpesviral replication and pathogenesis, as well as Rb family protein functional regulation during cell cycle progression and oncogenesis.

### Human herpesviruses

Prior to the genomics era, herpesviruses [104] were easily distinguishable because of their characteristic morphology [105,106]. Genome-containing icosahedral capsids are surrounded first by an amorphous layer of proteins termed the tegument, and subsequently by a lipid envelope. Viral glycoproteins in the virion envelope mediate fusion with, and entry into cells. Both the capsid and tegument are released into the cytoplasm. Tegument proteins modulate host cell processes even before the production of newly synthesized viral proteins from the infecting genomes, and help deliver the capsid along microtubules to the nuclear pore complex, where the genome is injected into the nucleus [107]. The linear, double-stranded DNA genome circularizes within the nucleus. Herpesvirus genomes [108,109] range in size from 120-kb and approximately 70 genes for Varicella Zoster Virus (VZV) to 235-kb and approximately 170 genes for human cytomegalovirus (HCMV). To start a productive, lytic replication cycle [110,111], a temporal and sequential cascade of immediate early (IE), early (E) and late (L) gene expression is initiated [104]. Viral DNA replication produces long concatamers that are packaged as unit length linear genomes into capsids within the nucleus. Newly formed capsids traverse the double nuclear envelope through an envelopment, de-envelopment pathway, acquire their tegument proteins and envelopes at cytoplasmic assembly sites derived form golgi membranes, and then exit the cell by the exocytosis of virion containing vesicles [104]. During latency, viral genomes are maintained as episomes (they generally do not integrate into host chromosomes), significantly fewer viral genes are expressed (the number varies between the different viruses), and no infectious virions are produced. Latent infections can be reactivated to allow for the new production of infectious virions decades after the primary infection.

**Figure 2:**
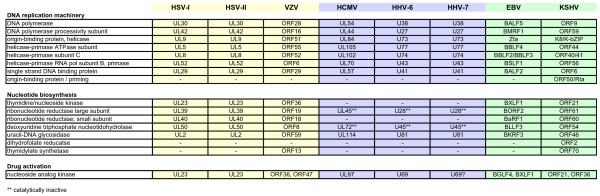
**Genes for DNA replication machinery and nucleotide biosynthesis encoded by the human herpesviruses**. Listed are the virally encoded genes involved or implicated in viral DNA replication (top), nucleotide biosynthesis (middle) and activation (by phosphorylation) of the nucleoside analogs (bottom) employed as anti-herpesviral drugs for the alpha- (yellow), beta- (blue) and gamma- (green) herpesviruses. Note the striking lack of nucleotide biosynthetic enzymes encoded by the betaherpesviruses.

**Figure 3:**
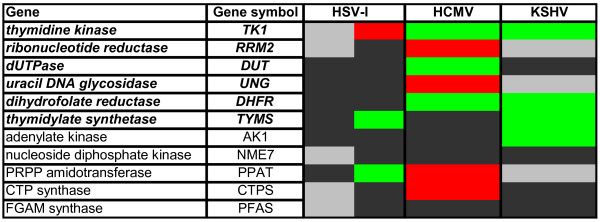
**Modulation of the steady-state levels of mRNAs for selected nucleotide biosynthetic enzymes by the different classes of human herpesviruses**. Microarray data for virus-infected cells was mined to illustrate the extent to which cellular nucleotide biosynthetic enzymes were activated upon infection with the indicated viruses. See text for a detailed discussion. HSV-1 infection of mouse embryonic fibroblasts at 4, 8, and 12 hours post infection (hpi) [317], left lane. HSV-1 infection of rat superior cervical ganglia neurons at 6, 12, and 24 hpi (Szpara and Enquist, personal communication), right lane. HCMV infection of human foreskin fibroblasts at 20, 24, and 48 hpi [316]. KSHV infection and reactivation of endothelial cells at 24, 37, and 48 hpi [315]. Red, up-regulated 2-fold or more in at least two out of three time points. Green, down-regulated 2-fold or more in at least two out of three time points. Black, any changes were less than 2-fold. Grey, not analyzed/not found. In cases where more than one probe set was present, fulfillment of above criteria by any one probe set was sufficient.

During lytic replication and in reactivating latent infections, herpesviruses must synthesize large quantities of viral DNA. The analysis of DNA content in herpesvirus-infected cells by flow cytometry indicates that cellular genome equivalents of viral DNA are produced in these cells. Therefore, herpesviruses must either rely on their own viral machinery for the enzymes required for nucleotide biosynthesis, metabolism, and polymerization (Fig. 2), or induce the accumulation of the cellular enzymes responsible for those same activities (Fig. 3). As many of those cellular enzymes are encoded by E2F-responsive genes, and as E2F-mediated gene expression is controlled in large part by the Rb proteins, this family of tumor suppressors is likely to be a critical target for the subset of herpesviruses that rely on cellular nucleotide biosynthetic enzymes and other DNA replication-related enzymatic functions for their replication.

**Figure 4:**
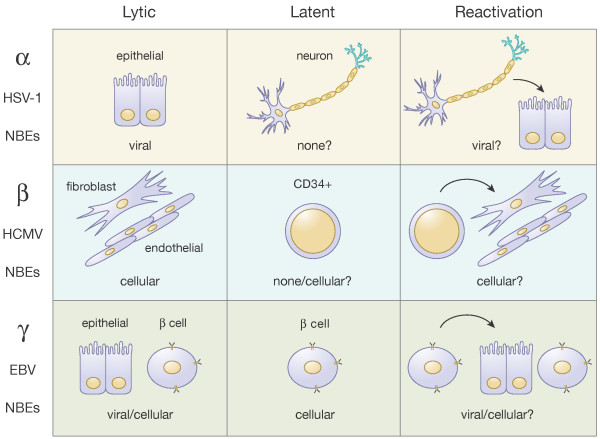
**Expression of nucleotide biosynthetic enzymes during the lytic and latent phases of different classes of human herpesviruses**. See text for details. **α**. During lytic infection HSV-1 does not induce the expression of cellular NBEs (nucleotide biosynthetic enzymes), and thus appears to rely on viral NBEs. It is unclear if the shutoff of host protein synthesis contributes to the absence of host NBE expression. During latency, viral genomes are not replicated and thus no NBEs appear to be required. Reactivation is assumed to be similar to lytic infection with a reliance on viral NBEs. **β**. During lytic infection HMCV robustly activates the expression of cellular NBEs, likely because it does not encode a full complement of viral NBEs. Reliance on NBEs during latency is not known because it is unclear if the viral genome replicates. However if viral genomic replication occurs, it is likely that cellular NBEs provide the needed nucleotides. Reactivation is assumed to be similar to lytic infection with a reliance on cellular NBEs. **γ**. During lytic infection EBV inactivates Rb and expresses its own NBEs, so both cellular and viral NBEs might be utilized. Note the shutoff of host protein synthesis during KSHV infection would appear to imply that viral NBEs play a more prominant role in KSHV lytic infection. During latency, viral proteins inactivate Rb and viral NBEs aren't expressed, implying a reliance on viral NBEs. Reactivation is assumed to be similar to lytic infection with a reliance on viral NBEs (for KSHV) or both viral and cellular NBEs (for EBV).

The eight herpesviruses that infect humans are classified into three families, the alpha-, beta-, and gammaherpesviruses (Fig. 4). Six of the eight human herpesviruses infect the vast majority of adults (the exceptions are HSV-2 and KSHV; see below for details). For each of these viruses, we will briefly describe the clinical manifestations of infection, discuss in detail the fates and functions of the Rb proteins in infected cells, and end with what we consider to be timely and relevant questions for future exploration.

### Alphaherpesviruses

The human alphaherpesviruses [112,113] include Herpes Simplex Virus Type 1 (HSV-1, HHV-1 (Human Herpesvirus 1)), HSV-2 (HHV-2), and Varicella Zoster Virus (VZV, HHV-3). Both primary and reactivated infections with HSV-1 typically cause oral lesions (cold sores), while HSV-2 infections generally cause genital lesions although each of these viruses can infect either location. Primary infection with VZV causes varicella (chicken pox) and reactivation of latent infections causes zoster (shingles). HSV-1 and VZV are very common infections, but HSV-2 infects only about 8% of the population [114]. Alphaherpesviruses carry out productive, lytic replication in epithelial cells, and establish latent infections in sensory neurons. Neuroinvasiveness and their relatively rapid lytic replication cycle are defining characteristics of the alphaherpesviruses.

### Herpes simplex virus type 1 and 2

Cells infected with HSV-1 accumulate in the G1 phase of the cell cycle [115-117]. Serum-arrested (G0) cells do not enter the S phase after infection, and G0 cells simultaneously infected and stimulated with serum also fail to enter the S phase [115-118]. In these HSV-1 infected, G1 arrested cells, the steady-state levels of Rb do not detectably change and the protein is found in a hypophosphorylated state bound to E2F proteins [116,118].

#### Inhibition of Rb phosphorylation in HSV-1 infected cells

The viral ICP27 protein (the 27^th ^infected cell protein observed) is required to prevent the phosphorylation of Rb in HSV-1 infected cells [119]. ICP27 is a multifunctional immediate-early protein that modulates gene expression at multiple levels including transcription, mRNA processing, and translation [120]. Along with the tegument-incorporated virion host shut off (VHS) protein (UL41, the 41st gene in the unique long region of the genome) that degrades mRNAs [121], ICP27 plays a role in the inhibition of host protein synthesis by inhibiting mRNA splicing [122-124] (this has little effect on viral gene expression because most herpesvirus genes are not spliced). Host shut off likely contributes to, but is not sufficient for the ability of ICP27 to inhibit Rb phosphorylation [119]. The ICP0 protein has been shown to arrest cell cycle progression [125,126] although the role it plays in Rb hypophosphorylation upon HSV-1 infection has not been examined. Likewise, the combined roles that ICP0 and ICP27 may play in the G1 arrest instituted by HSV-1 have not been resolved.

Infection with an ICP27-null virus results in Rb phosphorylation [119] although the kinase(s) responsible for this phosphorylation has not been identified. Likely candidates, however, would include the cellular kinase complexes composed of cyclin D/Cdk4,6 and cyclin E/Cdk2, as these complexes are known to phosphorylate Rb in uninfected cells. These kinases are inactive in wild-type HSV-1 infected cells for multiple reasons, such as the failure of cyclins to accumulate [115,117], the sequestering of Cdks in the cytoplasm [117], and the inhibition of preexisting cyclin/Cdk complexes by an unresolved mechanism that may be independent of the Cki proteins [117]. Further experiments performed in cells infected with ICP27 or ICP0-null HSV-1 could help define how Rb is regulated upon HSV-1 infection. It would be particularly interesting to determine if small molecule Cdk inhibitors such as roscovitine or flavopiridol have any effect on Rb phosphorylation in cells infected with these mutant viruses.

Curiously, one report indicated that Rb phosphorylation is induced by HSV-2 infection even though progression of infected cells into the S phase was inhibited [127], a situation analogous to HCMV infection (see below). Cyclin A/Cdk1 was implicated in the phosphorylation of Rb in HSV-2 infected cells [127]. A subsequent report was unable to confirm this, but found that both HSV-1 and HSV-2 did not induce Rb phosphorylation after infection of quiescent cells, and caused Rb dephosphorylation after infection of cycling cells [115]. A novel under-phosphorylated form of Rb in HSV infected cells (termed b') has been observed [115,117] although it is unclear whether this represents a novel site of phosphorylation or just an intermediately phosphorylated form. It is unlikely, however, that Rb-b' explains the different conclusions of the independent HSV-2 studies [115,127]. Thus, more work is needed to determine if Rb is regulated differently after infection with these two similar viruses and, if so, how that differential regulation affects viral replication, tropism, or pathogenesis.

The preponderance of evidence supports a model in which Rb is held in a hypophosphorylated (i.e. active) state in HSV infected cells, possibly because G1 cyclin/Cdk activity is low or absent. Interestingly, fibroblasts derived from mouse Rb -/- embryos (Rb-null MEFs) show no defects in supporting HSV-1 replication [128]. This indicates either that active Rb is not required for HSV-1 infection, or that other pocket proteins (p107 or p130) can compensate for Rb loss in these cells.

#### A critical role for p130 during HSV-1 infection

In quiescent (G0) cells, p107 is absent but upon serum stimulation its expression is induced as cells enter the S phase. HSV-1 infection coincident with serum stimulation inhibits the accumulation of the p107 protein [117]. In asynchronous cells infected with HSV-1, p107-E2F complexes were found to accumulate [118,129], a finding consistent with dephosphorylation such as that seen with Rb and p130 [117]. Similar to Rb, p107 function doesn't appear to be vital to HSV-1 lytic replication as p107-null MEFs support efficient viral replication [128].

HSV-1 infection inhibits the phosphorylation and subsequent degradation of the p130 protein that is observed as G0 cells enter the G1 phase, and causes a re-accumulation of hypophosphorylated p130 within infected G1 cells [117]. Contrary to what was observed with Rb and p107, p130 appears to be critical for lytic HSV-1 replication [128]. In p130-null MEFs, HSV-1 shows a 10- to 100-fold growth defect. A delay in IE gene expression was observed in these cells, as well as subsequent defects in viral DNA replication and late gene expression. The IE protein ICP0 was almost undetectable in these cells [128]. Because ICP0 is a critical regulator of HSV-1 lytic replication [130,131], it would be interesting to determine if ectopic expression of ICP0 corrected the HSV-1 replication defects observed in p130-null MEFs. HSV-1 infection failed to downregulate Cdk2 activity in p130-null MEFs [128]. When these cells were synchronized in early G1 (where Cdk2 activity is low), viral replication was partially rescued [117]. This is interesting because in addition to its ability to regulate E2F-mediated gene expression, p130 (and p107, but not Rb) can also function as a Cdk inhibitor [132-135]. Thus, the ability of the virus to downregulate G1 cyclin/Cdk activity through the action of the p130 protein (and not p130's effects on E2F) may be critical for efficient HSV-1 replication. However, one must remember that compensation between the Rb family members may occur in null MEFs [136]. So firm conclusions about the necessity of the individual pocket proteins for HSV-1 replication await independent confirmation.

A notable corollary to these results is that while the Rb proteins remain unphosphorylated in HSV-infected cells, the activity of kinases responsible for their phosphorylation, the Cdks, appears to be critical for HSV-1 replication [137]. Cdk activity is required for the efficient expression of viral genes [138]. Although the mechanism is not completely understood, transcriptional Cdks may contribute to HSV infection by regulating cellular RNA Polymerase II function [139] and/or enhancing the ability of the viral ICP0 protein to activate transcription [140]. Interestingly, the viral UL42 protein may serve to target the Cdks to their substrates in some of these putative phosphorylation events [141], either instead of, or in addition to, cellular cyclins.

### Varicella Zoster Virus

Cells infected with VZV do not progress into the S phase, but appear to arrest at the G1/S border [142]. Rb and p107 are not phosphorylated in VZV infected cells [142], similar to the results described above for HSV-1. The status of p130 in VZV infected cells has not been examined. However, unlike HSV-1, VZV infection induces the expression of cyclins D3, A, and B1 (but not cyclin E), and elevated Cdk2 and Cdk4 activity is observed [142,143]. Interestingly, this Cdk activity was observed in the presence of the Cki proteins p21 and p27. It is still unclear how these kinases remain active but do not phosphorylate Rb, a normal activity of these cyclin/Cdk complexes.

Small molecule Cdk inhibitors also inhibit VZV infection [144,145]. The broad spectrum Cdk inhibitor roscovitine reduces viral gene expression, viral DNA replication, and infectious virion formation [144], and a selective inhibitor of Cdk1 prevents the phosphorylation of the IE62 protein (immediate early protein of 62 kDa), a viral transcription factor [145]. Thus in a manner similar to HSV-1, Cdks seem to regulate viral gene expression, but not Rb protein phosphorylation, in VZV infected cells.

### Alphaherpesvirus summary

While Cdk activity clearly contributes to alphaherpesvirus infections, the Rb family proteins do not appear to be critical substrates of these kinases in cells infected with HSV-1, HSV-2, or VZV. These observations lead to two subsequent questions: why doesn't active Rb inhibit the replication of these DNA viruses, and what are the significant substrates of the active Cdks in alphaherpesvirus infected cells?

A potential answer to the first question is revealed upon a genomic comparison of the different human herpesviruses. While the cadre of DNA synthesis functions encoded by the three different classes of herpesviruses is similar, the alpha- and gammaherpesviruses encode considerably more nucleotide biosynthetic enzymes than the betaherpesviruses (Fig. 2). Thus, alphaherpesviruses (and perhaps gammaherpesviruses) may be less dependent on cellular E2F-responsive genes for viral DNA replication than the betaherpesviruses (see below) and therefore may not need to target Rb family members for inactivation (Fig. 1).

Candidates for important Cdk substrates in alphaherpesvirus infected cells include viral proteins and cellular RNA polymerase II. Determining whether the ''cell cycle'' Cdks (Cdk1, 2, 4, 6), the ''transcription'' Cdks (Cdk7, 9), or both are relevant targets for the broad spectrum Cdk inhibitors that decrease alphaherpesvirus replication may help define the critical targets of the kinases in infected cells. Also, determining how the Cdks recognize their targets could also be informative. Are cyclin/Cdk complexes relocalized during infection? Do viral proteins supplant cellular cyclins and re-direct Cdks to different substrates? Answers to these questions may help to resolve how Rb family members remain in their hypophosphorylated forms during alphaherpesvirus lytic infections even though some Cdks are active. Finally, the roles of Rb proteins and Cdks during latency of these viruses should be examined.

### Betaherpesviruses

The human betaherpesviruses [111,146-150] include Human Cytomegalovirus (HCMV, HHV-5), Human Herpesvirus 6A and 6B (HHV-6) and Human Herpesvirus 7 (HHV-7). These viruses are extremely common, with more than 90% of the population infected [147,149,151-153]. HCMV infections are mostly asymptomatic in healthy adults, but can cause severe disseminated disease in immunocompromised and immunosuppressed people [151]. HCMV is the leading infectious cause of birth defects, contributes to graft loss in transplant patients, is associated with atherosclerosis and restenosis, and becomes the major target of host cell-mediated immunity in older people, leading to immunosenescence [154]. A causative role in human malignancies has not been demonstrated, but HCMV is being evaluated as a cofactor for certain cancers, most notably glioblastoma. HHV-6 and HHV-7 are causative agents of exanthem subitum (''sudden rash''), a mostly benign disease in young children characterized by a fever and subsequent red rash (roseola) [150]. Collectively HHV-6 and HHV-7 are termed the Roseolaviruses. Like HCMV, reactivation of latent HHV-6 and HHV-7 infections in immunocompromised or immunosuppressed patients can be problematic. Betaherpesviruses are lymphotropic, but the true latent reservoirs of these viruses remain undefined. Lytic replication cycles are slow and can occur in multiple cell types *in vivo*, but are generally restricted to non-transformed human cells in culture. Persistent replication in salivary glands may be important for the natural transmission of these viruses to new hosts.

### Human cytomegalovirus

Infection of quiescent fibroblasts with HCMV results in their reentry into the cell cycle, progression through the G1 phase, and an eventual arrest at the G1/S border [155-161]. Infection of cycling cells also induces a G1/S arrest [160-162]. In G0 arrested cells, HCMV infection causes elevated levels of Rb which accumulate solely in the hyperphosphorylated form [156]. An examination of the very early stages of HCMV infection of quiescent fibroblasts indicated that hypophosphorylated Rb is first degraded, and then phosphorylated [163]. Both activities appear to be required for the absence of hypophosphorylated Rb, and the accumulation of hyperphosphorylated Rb during HCMV infection. Multiple HCMV proteins have been presented as candidate regulators of the Rb family proteins by degradation, phosphorylation or simple binding and inactivation. Furthermore, it is possible that HCMV can directly activate E2F-mediated gene expression independently of Rb. The individual viral proteins that modulate Rb, and the roles that they play in viral infection are discussed below.

#### Rb degradation in HCMV infected cells

The HCMV pp71 protein (a phospho-protein of 71 kDa) is a prominent component of the viral tegument [107,164] that binds to Rb and induces its degradation in a proteasome-dependent, ubiquitin-independent manner [165,166]. pp71 also binds and degrades p107 and p130 [166]. An LxCxD sequence in pp71 is required for Rb family degradation and for the ability of ectopically expressed pp71 to drive quiescent cells into the S phase of the cell cycle, as a mutant pp71 with an LxGxD motif (called C219G) failed to function in these assays [166]. pp71 can also accelerate progression through the G1 phase of the cell cycle by an unknown mechanism that is likely Rb-independent, because the LxCxD motif is not required for this activity [167]. Interestingly, pp71-mediated cell cycle stimulation does not induce apoptosis and pp71 is unable to cooperate with cellular or viral oncogenes to transform primary rodent cells *in vitro *[166,168], unusual properties for an Rb inactivating protein.

The early work on pp71 degradation of Rb was performed outside of the context of an HCMV infection, and in cell types non-permissive for HCMV infection. More recent experiments have addressed the role for this function of pp71 in HCMV infected, fully permissive fibroblasts *in vitro*. A virus expressing only the C219G mutant from of pp71 replicates as well as wild-type HCMV [169], indicating that at least in fibroblasts *in vitro*, Rb degradation by pp71 is not required for lytic replication, perhaps due to the multiple, redundant mechanisms HCMV uses to modulate the Rb-E2F pathway (see below). However, infection with this C219G mutant virus was used to demonstrate that pp71 is required for the degradation of hypophosphorylated Rb at very early times after HCMV infection [163]. pp71 is introduced into cells immediately upon infection and mediates the transient drop in the steady state levels of Rb that can be seen as soon as 2 hours after infection [163].

Although Rb degradation by tegument-delivered pp71 may not be required for lytic replication, degradation of another pp71 substrate, the Daxx protein, greatly enhances lytic replication [169-173]. Daxx is a transcriptional co-repressor that silences the HCMV major immediate early promoter (MIEP) [172,174,175]. The MIEP controls the expression of the viral IE proteins that, when expressed, commit the virus to the lytic replication cycle. By degrading Daxx, pp71 relieves this repression, facilitating IE gene expression and lytic replication. Because IE proteins, and an early protein (UL97) whose expression is activated by them, also regulate Rb (see below), pp71 appears to have both direct and indirect effects on the Rb-E2F pathway. Daxx degradation by pp71 is especially important during low multiplicity infections [169,170], and maintaining Daxx-mediated repression of the MIEP by preventing pp71 from degrading Daxx may contribute to the IE gene silencing that is observed when latent infections are established [176].

Interestingly, the substrates of pp71 (both Rb and Daxx) although initially degraded, re-accumulate at later times after infection [163,164,170,177]. It is presently unclear whether this is a result of an inhibition of pp71-mediated degradation, enhanced production of these pp71 targets, or both. Also unclear is the significance (and mechanism) of the uncommon ubiquitin-independent mode of proteasomal degradation mediated by pp71 [165,177]. Because pp71 can induce the degradation of its substrates when expressed alone in cells [165,170,177], no other viral proteins are required. However it is not known if cellular proteins (other than the proteasome) are required for pp71-mediated protein degradation. The importance of pp71-induced protein degradation to HCMV lytic infection and the uncommon method of that degradation make this an attractive target for the development of an inhibitory drug that may have potent antiviral activity but limited toxicity to uninfected cells.

#### Rb phosphorylation in HCMV infected cells

The Rb protein becomes hyper-phosphorylated as soon as 4 hours after HCMV infection of quiescent (G0) cells [156]. HCMV infection activates cyclin E- and cyclin B-dependent kinase activity [156,159] (but not cyclin D- or cyclin A-dependent kinase activity), and cyclin E/Cdk2 complexes are known to phosphorylate Rb. Thus it was surprising to find that small molecule inhibitors of the Cdks used at levels that completely inhibited serum induced Rb phosphorylation had no effect on Rb phosphorylation in HCMV infected cells [163]. Studies with additional inhibitors demonstrated that the activity of the HCMV UL97 protein kinase was absolutely required for Rb phosphorylation in HCMV infected cells [163]. UL97 directly phosphorylates Rb *in vitro*, and specifically targets multiple residues that, when phosphorylated, disrupt Rb-E2F and Rb/HDAC complexes, rendering Rb inactive [163]. Ectopic expression of UL97 drives quiescent cells into the S phase of the cell cycle [163], and recombinant HCMVs that express either no [178] or a catalytically inactive form of UL97 [163,179] fail to induce Rb phosphorylation [163,180]. Thus the HCMV protein kinase UL97 is necessary and sufficient for the phosphorylation and inactivation of the Rb protein.

UL97 is a serine-threonine kinase [179] that augments, but is not absolutely required for HCMV lytic replication in fibroblasts *in vitro *[178]. UL97 null viruses have a substantial (100-fold) [178] growth defect that is partially (10-fold) rescued by propagation on dividing cells [178]. Deletion of the UL97 gene or inhibition of UL97 kinase activity results in a 5- to 20-fold decrease in viral DNA replication [181]. One might predict that this defect may be due to lower levels of certain E2F-responsive genes involved in nucleotide biosynthesis in these cells, and experiments to address this hypothesis are currently underway in our laboratory. Virion assembly and egress are also adversely affected by the absence of UL97 kinase activity [181], perhaps resulting from defects either in tegument protein phosphorylation/localization [182,183], or nuclear lamina breakdown [184]. UL97 is also a key protagonist for the small arsenal of drugs available to treat HCMV infections. UL97 is required to phosphorylate and thus activate the ganciclovir family of antiherpesvirus drugs [185,186], and UL97 itself is the target of maribavir [187-189], a compound currently in phase III clinical trials for treatment of HCMV-associated disease. The mutually exclusive and antagonistic actions of these drugs [190] unfortunately prevent their simultaneous use in a combination therapy regimen.

UL97 phosphorylates Rb and drives cell cycle progression, functions which are carried out in uninfected cells by the Cdks. In fact, UL97 can be described as a functional ortholog of cellular Cdks because it rescues the cell cycle defect in yeast cells lacking Cdk activity [163]. Interestingly, UL97 appears to be an unregulated Cdk ortholog that is not subject to the normal control mechanisms that can be instituted to restrict cellular Cdk activity, such as the requirement for activation by CAK-mediated phosphorylation and cyclin binding, and the inhibition by a specific tyrosine phosphorylation or binding by the Ckis [163].

Without the need for cyclin binding, we wondered how UL97 was able to target Rb. Cellular cyclins have two sequence elements that could direct Cdks to phosphorylate Rb. The D-type cyclins have LxCxE motifs that bind in the Rb pocket domain, and all cyclins have a hydrophobic patch that interacts with RxL motifs in C terminus of the Rb protein. Interestingly, we found that UL97 contains both motifs. In fact, UL97 has three LxCxE motifs, although disruption of any individual site has minimal effects on Rb phosphorylation [180]. We are currently generating LxCxE and hydrophobic patch mutants to determine if these sequences direct UL97 to phosphorylate Rb.

#### Roles for viral IE proteins in modulating the Rb-E2F pathway

The HCMV Immediate Early-1 and -2 proteins (IE1 and IE2) are promiscuous transcription factors. IE1 is required for replication at low multiplicities of infection [191,192], and stimulates cell cycle progression, but only in p53-null or p21-null cells [193,194]. IE1, through its first 85 amino acids, interacts with the Rb family member p107 [195,196], but not with Rb [197], and relieves p107-mediated, but not Rb-mediated repression of an E2F-responsive reporter [195]. A single report a decade ago proposed that IE1 was a kinase that phosphorylated p107 and p130 (but not Rb) *in vitro *[198]. *In vivo* phosphorylation was not examined. That study identified a 23 amino acid region within IE1 containing homology to the ATP-binding sites of over 500 other kinases [198]. However, our computer searches have not revealed this homology. Furthermore, we have clearly shown that Rb is not phosphorylated in HCMV infected cells that express IE1 but do not express UL97, indicating that IE1 likely does not play a direct role in Rb phosphorylation during HCMV infection [163]. More experiments are needed to determine if IE1 and/or UL97 is required for p107 and/or p130 phosphorylation in HCMV infected cells.

IE2 is absolutely required for lytic infection [199], and has been reported to bind Rb both *in vitro *and *in vivo *[200-202]. Amino acids 290–390 of IE2 are required for Rb binding [201], and this binding is abrogated by cyclin A-induced phosphorylation of Rb [200]. This binding could contribute to Rb inactivation in combination with prior pp71-mediated Rb degradation and subsequent UL97-mediated Rb phosphorylation. Additionally, IE2 has been shown to bind directly to the cyclin E promoter [203], and IE2 mutants lacking the first 194 amino acids (but retaining the putative Rb-binding region) fail to activate transcription and are unable to stimulate the cell cycle [204]. Thus, IE2 modulation of the Rb-E2F pathway may actually bypass Rb and act directly on E2F-responsive promoters.

The IE1 and IE2 transcripts share exons 1–3 and thus are identical through their first 85 amino acids. Their subsequent sequences are different because of alternative splicing of the gene, with IE1 using exon 4 and IE2 using exon 5. Interestingly, a viral mutant lacking exon 3 (IE1 and IE2 amino acids 30–77) is viable, but has a severe growth defect, and importantly fails to fully activate the expression of cyclin E [205], an E2F-responsive gene. The defect in cyclin E gene activation was not rescued by ectopic expression of IE1, indicating that IE2 (and not IE1) is required for full activation of cyclin E in HCMV infected cells [205]. The expression of UL97 in cells infected with this virus has not yet been examined and thus this mutant virus may be defective in E2F-mediated gene expression for multiple reasons. Interestingly, IE2 also arrests cell cycle progression in early S phase by an unknown mechanism [206,207].

#### Role of Rb inactivation during HCMV replication and pathogenesis

During HCMV infection, Rb is inactivated and E2F-responsive genes are highly expressed [208]. Hypophosphorylated Rb is not found in HCMV-infected cells because it is first degraded by pp71 and then phosphorylated by UL97, and both of these phenomena should induce the expression of E2F-responsive genes. Expression of E2F-responsive genes also appears to be directly activated by IE2, independently of the Rb protein. Although IE1 has been shown to stimulate the cell cycle when exogenously over expressed (in p53 or p21 mutant cells), it does not appear to have a significant effect on cyclin E expression in the context of an HCMV infection of wild-type cells. It appears that HCMV encodes partially redundant mechanisms to ensure efficient Rb inactivation and robust E2F-responsive gene expression. The role of Rb inactivation during HCMV replication and pathogenesis is not yet known, but could be required for the accumulation of nucleotide biosynthetic and other enzymes involved in DNA replication that the virus could then usurp for the replication of its own genome. The roles of p107 and p130 during HCMV infection have not been extensively studied, although a novel p130-containing complex likely regulates cyclin E expression during HCMV infection [209].

### Human herpesvirus 6 and 7

We could find only one report of the effects of HHV-7 on cell cycle progression. In that study [210], primary or immortalized T cells infected at a low MOI (0.1 pfu/cell) were observed to display elevated DNA contents after ten days of infection similar to the 4n levels of DNA observed in cells in the G2 or M phase of the cell cycle. While the authors concluded that HHV-7 institutes a G2/M arrest, it is unclear if the newly synthesized DNA observed in these cells is viral or cellular. In fact, elevated levels of cyclin B were observed in cells with DNA contents corresponding to the G1, S, and G2/M phases of the cell cycle [210]. Levels of Cdk1 were also increased following HHV-7 infection [210]. No other cell cycle markers were analyzed. The results could be consistent with the authors' conclusion (a G2/M arrest), or may mimic the results seen with HCMV, where cells are arrested at the G1/S border but also express cyclin B, and where the DNA content increase in infected cells is attributed to viral, but not cellular DNA replication [156,159].

More work has examined the effects of HHV-6 infection on cell growth. T cells [211] or epithelial cells [212] infected with HHV-6B, and glial precursor cells infected with either HHV-6A or HHV-6B [213], stop dividing, rapidly cease synthesizing cellular DNA, and arrest with a G1/S (2n) DNA content. The G1/S arrest was clearly shown in glial precursor cells [213] by using a previously described method [214,215] in which the microtubule depolymerizing agent nocodazole is used to trap cycling cells in the G2/M phase, allowing for unambiguous quantitation of cells trapped in G1. While the levels of the p53 tumor suppressor are elevated in HHV-6 infected cells [211,212,216] (as they are during HCMV infection), p21 levels are not elevated [212,217], and the G1/S arrest appears to be p53-independent [212]. Cord blood mononuclear cells infected with HHV-6A (and to a lesser extent HHV-6B) showed significantly elevated levels of p53 and cyclin B, and a modest induction of cyclins A and E [217]. Similar to the HHV-7 study [210], the conclusion of a G2/M arrest based on the late accumulation of cells with a 4n DNA content is complicated by the inability to distinguish viral and cellular DNA by flow cytometry.

No studies that analyzed the ability of HHV-6 or HHV-7 to stimulate cell cycle progression or to modulate the Rb-E2F pathway at the molecular level could be found. Therefore, we compared the amino acid sequences of the HCMV proteins that modulate the Rb-E2F pathway (pp71, IE2, and UL97) to their HHV-6 and HHV-7 orthologs (U54, U86, and U69, respectively) in an attempt to predict how HHV-6 and HHV-7 may regulate progression through the G1 phase of the cell cycle. The pp71 orthologous U54 genes had no LxCxE motifs, indicating that if it does modulate Rb, it does so in a manner distinct from pp71. The IE2 orthologous U86 genes were found to be around 20% identical and 65% similar to IE2 within the regions of IE2 implicated in cell cycle induction (residues 1–194) and Rb binding (residues 290–390). Because small functional motifs within these regions have not been mapped, it is difficult to know whether this level of homology can indicate that conserved or divergent activities may be mediated by these protein domains. The UL97 orthologous U69 genes lacked a discernable hydrophobic patch, and contained only a single LxCxE motif that aligned with the first LxCxE motif in UL97. Interestingly, these proteins robustly phosphorylate Rb in transfected Saos-2 cells (Chad Kuny and Rob Kalejta, manuscript in preparation). Thus it seems likely that HHV-6 and -7 at least phosphorylate Rb through the action of their virally encoded protein kinase. Whether or not these viruses encode the multiple redundant functions of HCMV that modulate this pathway remains to be determined.

### Betaherpesvirus summary

Although cellular Cdks don't appear to play a role in the phosphorylation of Rb during HCMV infection, their activity is required for efficient viral replication as evidenced by reduced viral yields in the presence of a Cdk inhibitor such as roscovitine or a dominant-negative Cdk2 [218-221]. Thus, analogous to the alphaherpesviruses, Cdks play a significant role in viral replication despite their inability to phosphorylate Rb, and the relevant cellular and viral targets of the Cdks in HCMV-infected cells have yet to be identified. Although not a result of Cdk activity, the Rb protein is efficiently inactivated in HCMV infected cells, and E2F responsive genes are highly expressed. The conspicuous absence of almost all of the nucleotide biosynthetic enzymes encoded by the alpha- and gammaherpesviruses (Fig. 2) indicates the possibility that the betaherpesviruses are hyper-dependent on cellular enzymology for nucleotide synthesis and metabolism (see below).

In terms of pathogenesis, numerous studies have found HCMV, HHV-6, and HHV-7 genomes or proteins in cancerous cells or tissues [147,148,222]. However, the ubiquitous nature of these viruses means that such association studies need to be interpreted with caution. Certainly HCMV encodes multiple proteins that stimulate the cell cycle (Fig. 1) and that, theoretically, could have oncogenic potential. Sequence analysis and preliminary data indicate that at least one of the HHV-6 and HHV-7 orthologs of those proteins, the UL97 orthologous U69 protein kinases, are likely to have similar effects. In addition, HCMV encodes numerous anti-apoptotic proteins [223] and infection has been shown to prevent the death of some cancerous cells [224]. If any of these pro-proliferative or anti-apoptotic proteins were to be driving forces behind oncogenesis, their continued expression in tumors would appear to be essential to maintain the transformed phenotype. In addition, some HCMV proteins are mutagenic [225] and thus may cause genomic instability leading to cancer through a "hit and run" mechanism. Thus, multiple functions of these viruses could contribute to oncogenesis.

However, infection with betaherpesviruses does not transform cells *in vitro*. Furthermore, HCMV encodes many proteins that can arrest cell cycle progression, such as UL69 [226,227], IE2 [206,207] and UL117 (Yu, D. and Qian, Z. 2008 International Herpesvirus Workshop abstract). Furthermore, the HHV-6A U94 protein has been reported to suppress H ras-mediated transformation [228]. Many questions remain as to whether betaherpesvirues are opportunistic bystanders or propelling forces when they are found in human tumor samples, and if, in analogy to the papillomaviruses, there are "high risk" and "low risk" betaherpesviruses. The HCMV strains studied *in vitro *were isolated from patients with various manifestations of disease (e.g. tonsilitis, retinitis, congenital infections, transplant patients), but none that we know of were isolated from cancer patients. Comparisons of these strains to ones isolated from patients with potentially HCMV associated cancers may or may not reveal differences in the viral cell cycle regulatory proteins between strains. Furthermore, because most of the proteins with oncogenic potential are expressed only during lytic replication, it is unlikely that latently infected cells are transformed (as is the case for the gammaherpesviruses; see below), perhaps indicating that abortive or persistent infections may be linked to oncogenesis. To complement the epidemiological studies, translational approaches such as *in vitro *molecular analysis of betaherpesvirus infected cells isolated from human tumors could begin to answer the many confounding questions related to the potential direct role of these viruses in human cancers.

### Gammaherpesviruses

The human gammaherpesviruses [229-237] include Epstein Barr Virus (EBV, HHV-4) and Kaposi's Sarcoma Associated Herpesvirus (KSHV, HHV-8). EBV is common within the population (over 90% are EBV positive), infects B cells and epithelial cells, is transmitted orally, and causes infectious mononucleosis [229]. EBV is also associated with cancers, including B cell tumors such as Burkit's lymphoma and Hodgkin's lymphoma, and epithelial tumors such as nasopharyngial carcinoma [229,230,238]. In these cancers, EBV is maintained in a latent state. While most pathologies related to EBV are caused by latent infection, lytic EBV is associated with the proliferative disorder oral hairy leukoplakia in immunocompromised patients [229]. KSHV is uncommon in the general population (less than 7%, but some geographical areas have infection rates as high as 60%) infects B cells and endothelial cells, and is transmitted sexually. A latent KSHV infection is associated with Kaposi's Sarcoma, Primary Effusion Lymphoma (PEL), and a subset of Multicentric Castleman's Disease (MCD) [231,239]. The gammaherpesviruses are characterized by their tropism for lymphocytes, the association of their latent infections with human cancers, and the difficulty in modeling their lytic replication cycles *in vitro *[231].

### Epstein Barr virus

Lymphoblastoid cell lines (LCLs) develop when primary B-lymphocytes are infected *in vitro *with EBV [232,233]. LCLs are immortalized and transformed, so they proliferate and divide. Not surprisingly, they were found to have higher levels of Cdks and G1 cyclins compared to primary B-lymphocytes, and to harbor phosphorylated forms of Rb and p107 [240,241]. EBV infection in LCLs is latent. The viral genome is maintained as a circular episome and is replicated mainly by cellular proteins, although EBNA-1 (Epstein-Barr Nuclear Antigen – 1) is required for its replication and faithful partitioning during cell division [232,233,242]. In LCLs, EBNA-1, EBNA-2, EBNA-3a, EBNA-3c, EBNA-5 (EBNA-LP) and LMP1 (Latent Membrane Protein – 1) cooperate to induce and maintain the transformed phenotype [232,233]. A number of these proteins (and a few others) have been reported to affect the Rb-E2F pathway and are discussed below.

EBNA-5 does not have an LxCxE motif or hydrophobic patch, but has been reported to bind Rb in GST pull down experiments [243]. However, this protein was unable to counteract the repressive effects of Rb or p107 on a reporter construct under the control of a Gal4-E2F-1 fusion protein [244], so the relevance of Rb binding is unknown. In cooperation with EBNA-2, EBNA-5 can activate the expression of cyclin D2 when overexpressed in resting B cells stimulated with the viral gp350 envelope protein [245]. The ability of other EBV latent proteins to induce cyclin D2 expression under these conditions was not analyzed. More direct assays are required to determine if EBNA-5 can regulate the Rb-E2F pathway, and what role such putative modulation may have in the creation of LCLs upon EBV infection of primary B cells.

LMP-1 has also been reported to modestly induce cyclin D2 expression, perhaps indicrectly through the induction of the cMyc and AP1 transcription factors, and to maintain Rb in a hyperphosphorylated state in B cells treated with TGF-β[246,247]. Other EBV latent proteins were not analyzed for this function. Additional ways that LMP-1 may contribute to inducing E2F-mediated gene expression and cell cycle progression are by down regulating the expression of the Cki p16 [248] through inducing the nuclear export of the Ets2 transcription factor that induces p16 expression [249], and by causing the nuclear export of E2F-4 and -5 (the "inhibitor E2Fs") perhaps through disrupting their ability to bind Rb [249]. Because D-type cyclin expression is induced by growth factor signaling, it is straightforward to imagine how a membrane protein such as LMP-1 might activate cyclin D2 expression through activation of the signaling cascade involved in normal induction of cyclin D expression. It is more difficult to envision how such a protein may specifically regulate the nuclear export and complex formation of certain transcription factors, unless this is an indirect effect.

The EBNA-3C protein has also been implicated in modulating the Rb pathway. Although it lacks an LxCxE motif or a discernable hydrophobic patch, early work reported an interaction between *in vitro *translated and labeled EBNA-3C and GST-Rb [250]. In this same study, EBNA-3C also cooperated with activated ras to transform rodent cells. This transformation was insensitive to inhibition by over expression of p16 [250], leading the authors to speculate that inactivation of Rb, perhaps by inhibition of p16 or stimulation of cyclin D-dependent kinase activity was the mechanism through which EBNA-3C contributed to cellular transformation. A subsequent report showed that EBNA-3C was required for the continued proliferation of LCLs and for keeping the levels of both the p16 protein and transcript low [251]. This compelling study proposed that EBNA-3C may repress the transcription of p16, but provided no mechanism, and no indication that repression of p16 was necessary or sufficient for EBNA-3C induced proliferation of LCLs. A recent report proposed a p16-independent way in which EBNA-3C may modulate the Rb pathway. By translocating the mitochondrial protein MRS18-2 into the nucleus, EBNA-3C has been reported to facilitate MRS18-2 binding to Rb and disruption of Rb-E2F complexes [252]. The mechanism by which EBNA-3C could shuttle MRS18-2 to the nucleus remains unexplored.

Other means through which EBNA-3C may modulate the Rb pathway have also been proposed. EBNA-3C may increase cyclin A-dependent kinase activity by associating with cyclin A, disrupting its binding to the Cki p27, and leading to p27 degradation [253,254]. While the C terminus of EBNA-3C was required to render cyclin A insensitive to p27 [253], the same group subsequently found an N-terminal region of EBNA-3C (amino acids 130 to 159) bound more strongly to cyclin A (and bound to cyclins D1 and E), and inhibited cyclin A-dependent kinase activity [254]. Curiously, this same region (amino acids 140 to 149) was also implicated by this group in the EBNA-3C-mediated degradation of p27 [255] and Rb, but not p107 or p130, by recruitment of the Skp2 ubiquitin ligase complex [256]. It is unclear how this observation relates to the ability of hypophosphorylated Rb in uninfected cells to induce the degradation of Skp2 and thus result in cell cycle arrest by preventing Skp2-mediated degradation of p27 [257,258]. Because this small region of EBNA-3C may have multiple and possibly important effects on cell cycle progression, it is now critical to examine the role of this region of EBNA-3C in the context of an EBV infection.

The EBV genes expressed in LCLs are referred to as the latency III phenotype and can be expressed by EBV-positive cancers in immunocompromised patients [232,233]. Interestingly, in most natural latent EBV infections that lead to cancers in immunocompotent hosts, fewer genes are expressed. The only gene product implicated in Rb regulation that is consistently expressed in EBV positive tumors is LMP-1 [232,233]. It is likely that the extra latency III genes are initially expressed *in vivo* in all EBV transformed cells. However, the growth advantage they provide may be quickly outweighed by a propensity to permit immune detection and clearance, and thus are only consistently found in EBV transformed cells *in vitro* or in immunocompromised hosts. Therefore, in immune competent hosts, it is likely that an accumulation of additional cellular mutations (such as translocations that activate c-Myc expression) provide the growth or survival advantages required for transformation in the absence of latency III proteins. The required accumulation of these mutations along with immune surveillance may explain the low frequency with which natural infections lead to cancer as compared to the much higher *in vitro *transformation efficiency of EBV.

When latent EBV is induced to reactivate a productive lytic infection, the infected cells rapidly cease dividing and synthesizing cellular DNA, and arrest at the G1/S border with hyperphosphorylated Rb and elevated levels of cyclins E and A [259,260]. The two viral proteins that drive lytic reactivation, Z and R, may also play significant roles in these cell cycle effects. When expressed alone, the EBV Z protein has been shown to induce the expression of certain S phase genes, but also to arrest cell cycle progression in both the G1 and G2 phases [261-263]. These effects may be cell type specific. Thus Z appears to have similar activities to the HCMV IE2 protein that induces and then subsequently arrests cell cycle progression. The ability of Z to directly bind to Rb has not been shown. The EBV R protein does not have an LxCxE motif or a discernable hydrophobic patch, but still binds to Rb [264] and stimulates cell cycle progression [265]. In cells overexpressing R, low levels of Rb, p107 and p130 were observed [265] but the ability of R to degrade the Rb proteins was not examined. These cells also expressed high levels of E2F-1, and eventually died by apoptosis. In addition to the cellular E2F-responsive genes that likely contribute to viral DNA replication during EBV lytic replication, the viral DNA polymerase also appears to be an E2F-responsive gene [266].

Small molecule Cdk inhibitors were able to inhibit EBV lytic replication and viral gene expression [267]. In these inhibitor treated cells, Rb was found to be hypophosphorylated, perhaps indicating that cellular Cdks are responsible for phosphorylating Rb in lytically induced EBV infected cells. However, we have shown that the HCMV kinase UL97, and not the Cdks, phosphorylates Rb during HCMV lytic replication. Furthermore, our preliminary experiments indicate that the EBV ortholog of UL97, the BGLF4 protein, can also phosphorylate Rb when transfected into Saos-2 cells (Chad Kuny and Rob Kalejta, manuscript in preparation). Thus the small molecule Cdk inhibitors may prevent the expression of BGLF4 during EBV lytic reactivation, with BGLF4 (and not the Cdks) being directly responsible for the phosphorylation of Rb. More work is required to determine which kinase or kinases phosphorylate Rb during lytic EBV infection, and if the relevant targets of the small molecule Cdk inhibitors that prevent EBV lytic infection are the "cell cycle" Cdks or the "transcription" Cdks.

### Kaposi's sarcoma associated herpesvirus

Latent KSHV infections are studied *in vitro *either in cell lines established from PELs, or by infection of endothelial cells to create ''spindle cells'', similar to those observed in natural KS lesions. Cells latently infected with KSHV consistently express three proteins; LANA (latency associated nuclear antigen; ORF73), v-cyclin (viral cyclin; k-cyclin; ORF72), and V-FLIP (viral FLICE inhibitory protein; ORF71; K13) [268].

Although it lacks an LxCxE motif or a hydrophobic patch, LANA binds to and inactivates Rb, and cooperates with H-ras to transform rodent cells [269]. Proliferative diseases were also observed in transgenic mice expressing LANA from its endogenous promoter [270]. In addition to direct inactivation, LANA has also been reported to inactivate Rb through indirect mechanisms. By sequestering the GSK3β kinase in the nucleus, LANA expression leads to the stabilization of β-catenin, which in turn leads to the induction of cyclin D1 expression and the subsequent stimulation of G0 cells into the cell cycle [271]. LANA, and to a lesser extent v-cyclin, increases the levels of Id-1, perhaps by a post-transcriptional mechanism [272]. The Id proteins are naturally occurring dominant negative inhibitors of basic helix-loop-helix DNA binding transcription factors that are implicated in many processes such as the inhibition of differentiation and the stimulation of cell cycle progression [273]. However, because Id protein levels increase as cells progress through the cell cycle, it is unclear if LANA effects on Id-1 are direct or indirect, that is if they induce, or are induced by, cell cycle progression. Regardless of the mechanism(s), LANA expression has been clearly shown to activate a subset of E2F-responsive genes [274] presumably through Rb inactivation, because reporter assays and *in vitro *binding studies indicate that LANA is likely unable to inactivate either p107 or p130.

KSHV also encodes an ortholog of cellular cyclin D (v-cyclin) that is expressed during both lytic replication and latency [268,275-280] and phosphorylates Rb [276,281-283]. v-cyclin lacks the LxCxE motifs found in the D-type cyclins, but does have a hydrophobic patch that is highly conserved among herpesvirus-encoded cyclins [284]. It is not known whether the hydrophobic patch is required for Rb phosphorylation. Although it can bind to Cdk2, 4, 5, and 9, v-cyclin preferentially pairs with Cdk6 [276,281,282]. v-cyclin/Cdk6 complexes have an extended substrate range (compared to cyclin D/Cdk6) that includes targets of cyclin E and cyclin A [276,277,281,285-289]. In addition, v-cyclin is immune to a number of cellular control mechanisms that can attenuate the activity of cellular cyclin/Cdk complexes [290]. For example, v-cyclin lacks a destruction box so it is more stable than cellular cyclins [291], it is immune from inhibition by the Ckis [285,286,292], accumulates in the nucleus [293], and supports Cdk6 kinase activity in the absence of CAK phosphorylation [293,294]. Outside the context of a KSHV infection, v-cyclin can cooperate to transform rodent cells in culture and promote lymphoma formation in transgenic mice [295]. Through the phosphorylation and stabilization of p53 v-cyclin causes a G1 cell cycle arrest [296], so cell cycle stimulation and transformation is more readily observed in p53 mutant cells. Cells from latent KSHV infections or associated cancers, however, are not growth inhibited, potentially through the modulation of the p53 pathway by LANA [297]. Our preliminary evidence indicates that the KSHV-encoded kinase, the ORF36 protein, can also lead to Rb phosphorylation (Chad Kuny and Rob Kalejta, manuscript in preparation), indicating another potential mechanism for Rb inactivation during lytic replication.

Induction of lytic infection in PEL cell lines with TPA prevents them from entering the S phase [298] inducing an accumulation of cells with a G1 DNA content [299]. At least two lytic phase viral proteins seem to be involved in this G1 arrest. The K-bZIP protein (also known as RAP (replication associated protein) or K8), the structural and positional ortholog of the EBV Z protein, arrests cells in G1 [298,299]. Potential mechanisms for this arrest are the activation of p53 by direct binding [300], the stimulation of the transcription of the gene for the Cki p21 [298], and/or by directly binding and inhibiting cyclin E/A/Cdk2 complexes [299]. Interestingly, while K-bZip seems to share cell cycle functions with EBV Z, it is unable to induce lytic reactivation of latent KSHV infections [301]. This function seems to be confined to KSHV Rta, the ortholog of the EBV R protein [302,303]. The other lytic phase protein that has been shown to arrest PEL cells in G1, the viral G protein coupled receptor (v-GPCR, ORF74), likely does so through a p21-dependent mechanism [304]. This is intriguing in light of the fact that vGPCR is considered a transforming oncogene [305-309].

The viral cyclin is expressed during lytic infection as well [276,277]. Interestingly, the MGHV-68 cyclin is required for reactivation from latency [310]. Whether this is true or not for KSHV remains to be examined, but v-cyclin has been shown to phosphorylate different residues of p27 in latently and lyticaly infected cells [287], indicating that it may have important roles during both latency and lytic reactivation.

### Gammaherpesvirus summary

In contrast with the alpha- and betaherpesviruses there is a clear association of gammaherpesviruses with proliferative disorders including a number of cancers. Furthermore, both EBV and KSHV appear to encode proteins that modulate the Rb-E2F pathway, either directly or indirectly (Fig. 1). However, what appears to be an unambiguous case of cause and effect may not be so straightforward. EBV certainly encodes transforming proteins required for the maintenance of LCLs created *in vitro*. However, most of those are not expressed in the majority of naturally arising EBV positive tumors. So while these proteins can transform cells, and EBV does cause cancer, the Rb-inactivating viral proteins that may help initiate transformation are not always required for the maintenance of the transformed state. Such a view fits well with the ability of EBV to potentially activate cellular E2F-responsive genes by inactivating Rb (Fig. 1) while encoding a full complement of viral nucleotide biosynthetic enzymes (Fig. 2).

Similarly, while KSHV encodes Rb inactivating proteins (Fig. 1), a provocative study found that the Rb pathway could be reconstituted in KSHV infected PEL cells by expressing the Cki p16 [311]. The six PEL cell lines and four PEL tumor samples tested in this study were all found to be deficient for p16. Furthermore, when PEL cells were transduced with a recombinant adenovirus expressing p16, they arrested in the G1 phase of the cell cycle, and this arrest required the presence of the Rb protein [311]. Thus, even though PEL cells express LANA and v-cyclin, additional mutations (such as loss of p16) appear to be required to fully inactivate the Rb pathway. So even for the gammaherpesviruses, viral infection may not be sufficient for the complete inactivation of the Rb pathway that is required for carcinogenesis.

Interestingly, although Rb inactivating proteins such as v-cyclin are also expressed during lytic KSHV infection, this is unlikely to result in an increase in cellular E2F gene product accumulation. The KSHV-encoded SOX (shut-off and exonuclease; ORF37) protein mediates a broad shut-off of host gene expression [312-314] by causing the rapid turnover of more than 75% of host transcripts, while only 2% of host transcript levels increase in response to KSHV infection [315]. KSHV is likely immune to potetially negative effects of the downregulation of E2F responsive genes because it encodes the most nucleotide biosynthetic enzymes of any herpesvirus (Fig. 2).

### Nucleotide biosynthetic enzymes (NBEs) and human herpesvirus infections

What effects do the many different ways that the human herpesviruses regulate the Rb protein (to inactivate it or keep it active) have in the context of viral infection? Rb controls the E2F transcription factors, and many E2F-responsive genes are involved in DNA replication, a process required by all human herpesviruses to replicate their own genomes. But all the human herpesviruses seem to encode most of the enzymology required for the actual process of DNA synthesis (Fig. 2). So if cellular helicases, primases, and polymerases aren't needed for viral genome replication, than what cellular factors, if any, are?

We hypothesize that the nucleotide biosynthetic enzymes (NBEs) represent the key Rb-E2F-regulated gene products of interest to the human herpesviruses. However, not all human herpesviruses appear to have an equal interest in these genes. As alluded to earlier, the alpha- and gamaherpesviruses encode between five and seven enzymes involved in nucleotide metabolism, whereas the betaherpesviruses encode only one activate enzyme (Fig. 2). Not surprisingly, by mining published [315-317] and unpublished (Szpara and Enquist, personal communication) microarray studies, we found that a select set of cellular NBEs is elevated in HCMV infected cells, but not in HSV-1 or KSHV-infected cells (Fig. 3).

We selected a set of eleven cellular NBEs to analyze based on two criteria. First, we picked six cellular enzymes (TK1, RRM2, DUT, UNG, DHFR, TYMS) that have a virally encoded counterpart in at least one human herpesvirus. The second criteria was that the NBE was present on the arrays used to analyze changes in the expression patterns of cellular genes after infection with at least two of the three viruses (HSV-1, HCMV, and KSHV) for which we could access the primary data. There were five genes in this category (AK1, NME7, PPAT, CTPS, PFAS). We analyzed the available microarray data to determine if the expression level of these genes changed after viral infection.

For KSHV, we found that during lytic infection, none (0/11) of the cellular NBEs analyzed were upregulated (Fig. 3). In fact, many were downregulated, likely due to the action of the viral SOX protein. KSHV can likely succeed without the need of cellular NBEs because it encodes the most viral NBEs of any human herpesvirus (Fig. 2). We could not find a microarray analysis of latently infected KSHV cells, but we would expect to see upregulated expression of the cellular NBEs because the viral NBEs are not expressed during latency. We could not access the data for the only EBV study we found in the literature[318].

We found a similar absence of upregulated cellular NBEs during lytic (1/11) infections of HSV-1 (Fig. 3). These results aren't surprising because HSV-1 encodes many viral NBEs that are expressed during lytic infection and encodes a nuclease (VHS) that can degrade cellular mRNAs. We did not analyze an HSV-1 latency microarray study. However, HSV-1 establishes latency in non-dividing neurons and thus doesn't need to replicate its genome to high levels and we suspect cellular NBE levels to be low in latently infected neurons. We could not find a microarray analysis of HSV-2 or VZV infected cells.

In contrast, HCMV infection upregulated more (4/11) of the cellular NBEs during lytic infection (Fig. 3). Both the promoter and enzymatic activity of one enzyme not upregulated in the microarray study, DHFR, was found to be increased in HCMV infected cells [319,320], and thus we could set the activated fraction even higher (5/11). Furthermore, the virus may not need the activity of two of the other enzymes because the UL97 protein that phosphorylates the nucleoside analog ganciclovir[185,186] may be able to phosphorylate other nucleosides, and thus may functionally replace TK1 and NME7. HCMV likely relies on cellular NBEs because it encodes only one catalytically active viral NBE (Fig. 2). This was surprising as HCMV has the largest genome and the most genes of the human herpesviruses. We could not find microarray analysis of latent HCMV, HHV-6, or HHV-7 infections. What is not readily apparent is why or how the alpha- and gammaherpesviruses evolved to encode most of their own NBEs, while the betaherpesviruses seem to rely heavily on cellular enzymes. An intriguing hypothesis is that, by evolving multiple mechanisms to inactivate Rb, HCMV and perhaps betaherpesviruses in general may have eliminated their need to encode their own NBEs. The fact that betaherpesviruses each encode two catalytically inactive NBEs seems to lend credence to this theory. It would be interesting to determine if the expression of a full cadre of virus NBEs would obviate the need for some of the Rb inactivating proteins encoded by HCMV.

## Conclusion

In addition to inducing the expression of cellular NBEs, there appear to be two other reasons why herpesviruses may modulate the Rb-E2F pathway. First, by manipulating this pathway, they can synchronize infected cells in a cell cycle position that favors the efficient replication of their DNA genomes, and by extension, their productive lytic replication cycles. Such synchronization appears to require both cell cycle stimulatory and arrest functions. This favorable cell cycle position can be thought of as the G1 phase for all the herpesvirus classes. G1 arrest presumably is beneficial because preventing the replication of the cellular genome allows for the unencumbered utilization of DNA precursors and replication enzymes for the sole production of viral genomes. While this hypothesis for the presumed benefit that cell cycle arrest seen during herpesvirus lytic infections provided for these viruses is commonly invoked (and makes perfect sense), there is surprisingly little direct evidence to support it. It is known that replication of HCMV, the largest and slowest human herpesvirus, is delayed and decreased by an IE2 point mutant that fails to arrest cell cycle progression [321]. However it is unclear if the inability to arrest the cell cycle is the only deficiency shown by this mutant allele of this essential HCMV protein. Furthermore, as herpesviruses such as HSV-1 and EBV have smaller genomes and replicate much faster, it might be expected that competition for nucleotides would have a quantitatively smaller effect on these viruses. More work is needed to determine why G1 appears to be a favored cell cycle position for herpesvirus lytic replication.

The second reason why herpesviruses may modulate the Rb-E2F pathway is to facilitate cell division and thus the expansion of the reservoir of latently infected cells. While lytic replication can rapidly increase the number of infected cells within a host, and the progeny virions that are created can mediate transmission to new hosts, lytically-infected cells are short-lived and subject to intrinsic, innate, and adaptive immune defenses. Latency permits the long term persistence of infected cells that may be less visible to the immune system. If latently infected cells proliferate, or could be induced to proliferate, the reservoir of such cells could be expanded.

Thus, both lytic and latent infections of herpesviruses could conceivably benefit from modulation of the host cell cycle in general and the Rb-E2F pathway in particular. Below we summarize the data presented above for a representative member of each class of human herpesviruses (please see the companion figure, Fig. 4).

### HSV-1, an alphaherpesvirus

#### Lytic replication

Cell cycle arrest functions are expressed (ICP0, ICP27), but no cell cycle stimulatory functions are expressed. Cells arrest in early G1 phase. The expression of many virally-encoded nucleotide biosynthetic enzymes (Fig. 2) likely renders HSV-1 relatively insensitive to changes in E2F-mediated gene expression.

#### Latency

Cell cycle effects in latently infected cells are unknown, but because latency is established in terminally differentiated sensory neurons and few viral genes are expressed, any effects would be expected to be minimum. The non-dividing nature of the latent reservoir appears to render HSV-1-independent of E2F-mediated gene expression during latency. Sensory neurons would seem to be a non-renewable resource, so reactivations would either need to be non-cytolytic, or very judiciously initiated. The expression of viral nucleotide biosynthetic enzymes during reactivation may allow for lytic replication without cell cycle induction, perhaps improving the chances for the survival of the latently infected neuron after lytic reactivation.

### HCMV, a betaherpesvirus

#### Lytic replication

Cell cycle arrest (UL69, IE2, UL117) and stimulatory (pp71, IE1, IE2, UL97) functions are expressed. Infected cells are arrested at the G1/S border. The absence of many nucleotide biosynthetic enzymes encoded within the HCMV genome (Fig. 2) appears to make this virus dependent upon cellular E2F-responsive genes for efficient lytic replication.

#### Latency

Cell cycle effects during latent HCMV infections have not been examined. However, because few if any genes are expressed, and because transformation or immortalization of latently infected cells has not been demonstrated, it is unlikely that latency modulates the cell cycle. The absence of many HCMV-encoded nucleotide biosynthetic enzymes implies that reactivation requires cellular E2F-mediated gene expression. Whether this leads to death of the reactivated cell is unclear. Because the undifferentiated cells that likely harbor latent virus would appear to be a renewable resource, the eventual death of the reactivating cell may have few consequences for the formation of lifelong latency except for the need for continual reseeding of the latent reservoir.

### EBV, a gammaherpesvirus

#### Lytic replication

Cell cycle arrest (Z) and stimulatory (R) functions are expressed. Infected cells arrest in late G1 phase. The expression of many EBV-encoded nucleotide biosynthetic enzymes (Fig. 2) likely renders EBV relatively insensitive to changes in E2F-mediated gene expression.

#### Latency

Cell cycle stimulatory functions are expressed in type I (LMP-1) and type III (LMP-1, EBNA-3C, EBNA-LP) latency, but cell cycle arrest functions are not. Latently-infected primary B cells are immortalized and transformed. They proliferate and divide, and so the virus must replicate and partition its genome to maintain the latent reservoir. The lack of expression of virally-encoded nucleotide biosynthetic enzymes during latency appears to make EBV reliant upon E2F-mediated cellular gene expression for viral DNA replication. Note that during latency, the amount of viral DNA replication (which is mediated by host cell polymerases) is negligible compared to the amount of host cell DNA being synthesized (in contrast to what happens during lytic infection). Because the B cells that harbor latent virus represent a renewable resource, the eventual death of the reactivating cell may have little effect on the ability of EBV to establish lifelong latency. Because latently infected cells can expand in number, a continual reseeding of the latent reservoir may not be necessary.

## Competing interests

The authors declare that they have no competing interests.

## Authors' contributions

AJH and RFK contributed to the discussion and preparation of this manuscript.
